# A New Phenolic Glycoside from *Chamaecyparis obtusa* var. *breviramea* f. crippsii

**DOI:** 10.3390/molecules18011255

**Published:** 2013-01-18

**Authors:** Yu-Mei Zhang, Jian Xu, Lin Xiao, Guang-Zhi Zeng, Zhang-Hua Sun, Ning-Hua Tan

**Affiliations:** 1 State Key Laboratory of Phytochemistry and Plant Resources in West China, Kunming Institute of Botany, Chinese Academy of Sciences, Heilongtan, Kunming 650201, China; 2 Xishuanbanna Tropical Botanical Garden, Chinese Academy of Sciences, Xuefu Road 88#, Kunming 650223, China; 3 Key Laboratory of Ministry of Education on Traditional Chinese Medicine Resource and Compound Prescription, Pharmacy Faculty, Hubei University of Chinese Medicine, West Huangjiahu Road, Wuhan 430065, China; 4 Department of Plant Sciences, Agricultural College, Guangxi University, East Daxue Road, Nanning 630004, China

**Keywords:** *Chamaecyparis obtusa* var. *breviramea* f. crippsii, phenolic compounds, cytotoxicity

## Abstract

A new phenolic glycoside, 3-methoxyphenol 1-*O*-*α*-L-rhamnopyranosyl-(1→6)-*O*-*β*-D-glucopyranoside (**1**), was isolated from the 90% acetone extract of the branches and leaves of *Chamaecyparis obtusa* var. *breviramea* f. crippsii along with another 10 known phenolics **2**–**11**. Their structures were determined mainly by means of MS, 1D- and 2D-NMR data. Cytotoxicities of compounds **3** and **5**–**11** were tested on BGC-823, Hela and A549 cancer cell lines, the results showed that compound **8** was bioactive and its IC_50_ values were 6.9, 29.7 and 52.9 μM, respectively.

## 1. Introduction

There are six speciesin the genus *Chamaecyparis*, which are mainly distributed in North America, Japan, and Taiwan [1]. *Chamaecyparis* plants have been found to be rich sources of monoterpenes [2], sesquiterpenoids [3], diterpenes [2,4] and lignans [5,6,7], some of which have shown antitumor, antimalarial and antibacterial activities [8,9,10]. *Chamaecyparis obtusa* (Sieb. et Zucc.) Endl. var. *breviramea* f. crippsii is a cultivated variety of *C. obtusa* [11]. According to the literature, no chemical constituents of this plant has been reported until now. As part of serial investigations on the Cupressaceae and in order to seek more novel bioactive compounds, we carried out an extensive chemical study on *C. obtusa* var. *breviramea *f. crippsii. In this paper, we report the isolation and structure elucidation of a new phenolic glycoside **1**, together with ten other known phenolics **2**–**11** from the branches and leaves of *C. obtusa* var. *breviramea *f. crippsii, in addition to a screening of their cytotoxicity.

## 2. Results and Discussion

**Figure 1:**
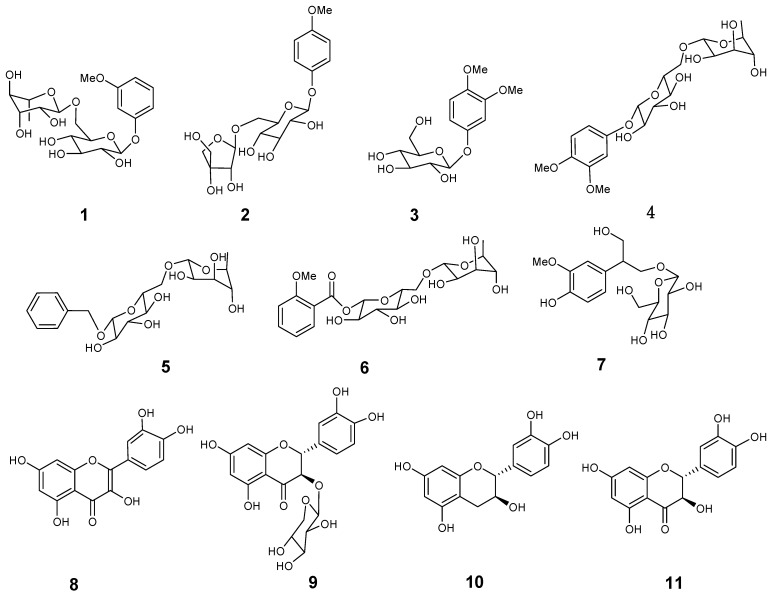
Structures of compounds **1**–**11**.

The *n*-BuOH fraction of the branches and leaves of *C. obtusa* var. *breviramea *f. crippsii was subjected to column chromatographies on silica gel, Sephadex LH-20 and MCI, and preparative HPLC, to afford a new phenolic glycoside **1**, together with the 10 known phenolics **2**–**11**, which were identified by comparison of spectra data with the reported literature values (Figure 1).

Compound **1** was obtained as a colorless amorphous solid. It possessed the molecular formula C_19_H_28_O_11_ according to HRTOFMS ([M+Cl]^−^ peak at *m/z* 467.1313, calc. 467.1320), which was confirmed by the ^13^C-NMR spectrum. The IR spectrum of **1** showed absorption bands for OH (3423 cm^−1^) and aromatic (Ph) groups (1596 cm^−1^). Its UV spectrum revealed the presence of aromatic (Ph) groups (200, 220, 273 nm). The ^1^H and ^13^C spectra (see Table 1) showed the presence of a *m*-substituted aromatic group (*δ*_C_ 147.9 (C-1), 124.2 (C-2), 150.8 (C-3), 113.7 (C-4), 122.3 (C-5), 118.2 (C-6); *δ*_H_7.03 (d, *J* = 2.9 Hz, H-2), 7.03 (m, H-4), 6.95 (m, H-5), 7.16 (d, *J* = 6.6 Hz, H-6)), a *β*-D-glucopyranose (*δ*_C_ 102.7 (C-1'), 74.9 (C-2'), 77.9 (C-3'), 71.6 (C-4'), 76.9 (C-5'), 67.7 (C-6'); *δ*_H_4.86 (d, *J* = 6.3 Hz, H-1'), 3.50 (t, *J* = 7.6 Hz, H-2'), 3.48 (t, *J* = 7.6 Hz, H-3'), 3.40 (t, *J* = 7.6 Hz, H-4'), 3.54 (dt, *J* = 6.8, 1.5 Hz, H-5'), 4.02 (dd, *J* = 9.0, 1.5 Hz, H-6'a), 3.62 (dd, *J* = 9.0, 5.1 Hz, H-6'b)), an *α*-L-rhamnopyranose [*δ*_C_ 102.2 (C-1''), 72.2 (C-2''), 74.0 (C-3''), 72.4 (C-4''), 69.8 (C-5''), 18.0 (C-6''); *δ*_H_4.72 (d, *J* = 1.2 Hz, H-1''), 3.83 (dd, *J* = 2.8, 1.4 Hz, H-2''), 3.70 (dd, *J* = 8.0, 2.8 Hz, H-3''), 3.37 (t, *J* = 8.0 Hz, H-4''), 3.66 (m, H-5''), 1.22 (d, *J* = 5.2 Hz, H-6'')], and a methoxy (*δ*_c_ 56.6), which suggested that compound **1** was a phenolic glycoside. The ^1^H- and ^13^C-NMR data of **1** (see Table 1) were a close match to those of itoside I [12].

**Table 1:** ^13^C- and ^1^H-NMR spectral data of compound **1** (*δ* in ppm, *J* in Hz).

Position	δ_C_	δ_H_		δ_C_	δ_H_
1	147.9		4'	71.6	3.40 (t, 7.6 Hz)
2	124.2	7.03 (d, 2.9 Hz)	5'	76.9	3.54 (dt, 6.8, 1.5 Hz)
3	150.8		6'	67.7	4.02 (dd, 9.0, 1.5 Hz) 3.62 (dd, 9.0, 5.1 Hz)
4	113.7	7.03 (m)	1''	102.2	4.72 (d, 1.2 Hz)
5	122.3	6.95 (m)	2''	72.2	3.83 (dd, 2.8, 1.4 Hz)
6	118.2	7.16 (d, 6.6 Hz)	3''	74.0	3.70 (dd, 8.0, 2.8 Hz)
OMe	56.6	3.88 (s)	4''	72.4	3.37 (t, 8.0 Hz)
1'	102.7	4.86 (d, 6.3 Hz)	5''	69.8	3.66 (m)
2'	74.9	3.50 (t, 7.6 Hz)	6''	18.0	1.22 (d, 5.2 Hz)
3'	77.9	3.48 (t, 7.6 Hz)			

^13^C- (125 MHz) and ^1^H- (500 MHz) in CD_3_OD.

**Figure 2:**
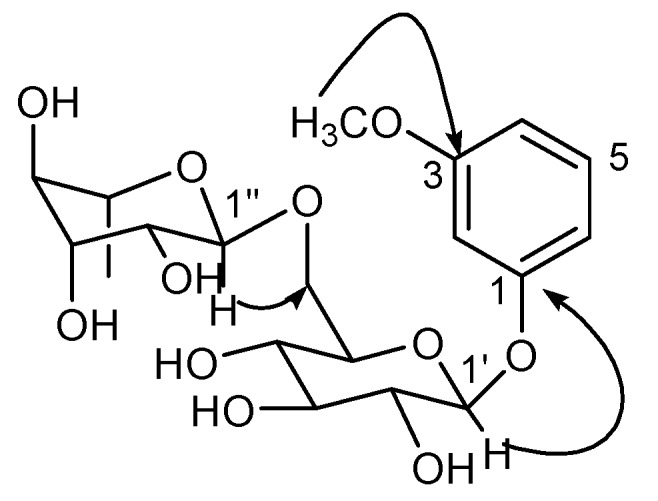
Key HMBC correlations of compound **1**.

In the HMBC spectrum of **1** (Figure 2), the correlation from the anomeric proton 4.72 (d, *J* = 1.2 Hz, H-1'') to the methine at 67.7 (C-6') indicated that the linkage between the *α*-L-rhamnopyranose and the *β*-D-glucopyranose was C-1''→C-6'. The cross peak between *δ*_H_4.86 (d, *J* = 6.3 Hz, H-1') and *δ*_C_147.9 (C-1) suggested that the substituted site of the *β*-D-glucopyranose on the phenolic aglycone was C-1'→C-1. The correlation between *δ*_H_3.88 (s, OMe) and *δ*_C_150.8 (C-3) indicated that the OMe was located at C-3. Thus, the structure of **1** was identified as 3-methoxyphenol 1-*O*-*α*-L-rhamnopyranosyl-(1→6)-*O*-*β*-D-glucopyranoside.

Compounds **2**–**11** were identified as 4-methoxyphenol 1-*O*-*β*-D-apiofuranosyl-(1→6)-*O*-*β*-D-glucopyranoside (**2**) [13], 3,4-dimethoxyphenyl-1-*O*-*β*-D-glucopyranoside (**3**) [14], 3,4-dimethoxyphenyl 1-*O*-*α*-L-rhamnopyranosyl-(1→6)-*O*-*β*-D-glucopyranoside (**4**) [15], 7-*O*-benzyl-*α*-L-rhamnopyranosyl-(1→6)-*O*-*β*-D-glucopyranoside (**5**) [16], 7-*O*-*α*-L-rhamnopyranosyl-(1→6)-*O*-*β*-D-glucopyranoside of methyl salicylate (**6**) [17], 1-*O*-*β*-D-glucopyranosyl-2-(3-methoxy-4-hydroxyphenyl)-propane-1,3-diol (**7**) [18], quercetin (**8**) [19], (+)-taxifolin-3-*O*-*β*-D-xylopyranoside (**9**) [20], (+)-catechin (**10**) [21] and (+)-taxifolin (**11**) [22] by comparison of their data ([α], MS and NMR) with those in the literature.

In the primary bioactivity test, the methanol extract of this plant showed cytotoxicities on the cancer cell lines A549, BGC-823, Du145 and MDA-MB-231 with IC_50_ values of 0.94, 1.07, 0.95 and 0.96 μg/mL, respectively. In order to find the cytotoxic constituents, among the **11** compounds obtained and identified from the *n*-BuOH fraction of this plant, compounds **3** and **5**–**11** were tested for cytotoxicity against the Hela, BGC-823 and A549 cancer cell lines, and only compound **8** showed cytotoxicity, with IC_50_ values of 6.9, 29.7 and 52.9 μM, respectively.

## 3. Experimental

### 3.1. General

Optical rotations were measured at 17 °C on a Horiba SEAP-300 polarimeter. IR spectra were obtained on a Bio-Red FTS-135 spectrophotometer. UV spectra were measured on a 2401PC spectrophotometer. NMR spectra were recorded on a Bruker AM-400 or DRX-500 spectrometer, using TMS as an internal standard. ESIMS spectra were obtained on a VG Autospec-3000 spectrometer. HPLC was performed using an Agilent 1100 autopurification system equipped with a DAD detector (190–950 nm). Precoated silica gel plates (Meijing, China) were used for TLC. Detection was done by spray plates with 8% anisaldehyde-sulfuric acid, followed by heating.

### 3.2. Plant Material

Branches and leaves of *C. obtusa* var. *breviramea* f. crippsii were collected from Kunming Botany Garden, Yunnan Province, People’s Republic of China, in August 2010. It was identified by Associated Prof. Zhong Shu Yue from Kunming Institute of Botany, Chinese Academy of Sciences.

### 3.3. Cytotoxicity Activity Assay

The human tumor cell lines Du145 (prostate carcinoma) and MDA-MB-231 (breast carcinoma), BGC-823 (gastric carcinoma), Hela (cervical carcinoma) and A549 (non-small cell lung carcinoma) were bought from the Chinese Academy of Medical Sciences. The cytotoxicity assays were performed according to a published procedure [23].

### 3.4. Extraction and Isolation

The powdered air-dried branches and leaves (12.5 kg) of *C. obtusa* var. *breviramea* f. crippsii were extracted with 90% acetone (3 × 20 L) at room temperature and then concentrated under reduced pressure. The concentrated acetone extract (860 g) was dissolved in hot water and extracted with petroleum ether, EtOAc and *n*-BuOH, respectively, to afford a 250 g petroleum ether fraction, a 110 g EtOAc fraction, a 210 g *n*-BuOH fraction and a 284 g water fraction. The *n*-BuOH fraction was purified by column chromatography (CC) on silica gel eluting with CH_2_Cl_2_/MeOH (9:1–0:1), to give sub-fractions 1–9. Sub-fraction 6 was respectively purified by CC and eluted with CH_2_Cl_2_/MeOH (8.5:1.5–7:3), MCI (MeOH/Water: 1:9–6:4), Sephadex LH-20 (MeOH/Water: 7:3), and then preparative HPLC using a Sunfire C-18 column (250 × 21.2 mm, 5 μm) with a mobile phase consisting of MeOH-Water (1.5:8.5–4:6) to afford **1** (11 mg), **2** (8 mg), **3** (26 mg), **4** (7 mg), **5** (21 mg), **6** (17 mg) and **7** (35 mg). Sub-fraction 2 was purified by CC and eluted with CH_2_Cl_2_/MeOH (9:1–8:2) to afford **8** (35 mg), **9** (24 mg), **10** (48 mg) and **11** (31 mg).

*3-Methoxyphenol 1-O-α-L-rhamnopyranosyl-(1→6)-O-β-D-glucopyranoside* (**1**): Colorless amorphous solid. m.p. 112–114 °C. [α]^22^_D_ = −48.9 (*c* = 0.28, MeOH). IR ν_max_ cm^−1^: 3423, 2928, 1676, 1596, 1504, 1257, 1068; UV *λ*_max_ (logε): 200 (4.13), 220 (3.85), 273 (3.26). ^1^H- and ^13^C-NMR spectral data: see Table 1. ESIMS *m/z*: 467 [M+Cl]^+^. HRTOFMS *m/z*: 467.1313[M+Cl]^+^ C_19_H_28_O_11_Cl (calc. for 467.1320).

## 4. Conclusions

This work was part of a series of investigations on antitumor compounds from Cupressaceae plants. Compound **1** was found to be a new phenolic glycoside, and the other ten compounds were found for the first time in *C. obtusa* var. *breviramea* f. crippsii. The random cytotoxic screening results showed that the significant cytotoxicity of the methanol extract was not caused by these simple phenolics isolated from the *n*-BuOH fraction, so the sesquiterpenes, diterpenes and podophyllotoxin derivatives isolated from the petroleum ether and EtOAc fraction might be responsible for the strong cytotoxicity of the crude methanol extract, which will be extensively investigated and reported in future work.
